# Urbanicity is associated with an earlier age at onset of psychosis: findings from a first-episode psychosis cohort

**DOI:** 10.1192/j.eurpsy.2026.10461

**Published:** 2026-07-31

**Authors:** H. Marín Mateos, F. Gotor-Sanchez-Luengo, G. Lahera Forteza, A. Lopez Diaz

**Affiliations:** 1 Mental Health Clinical Management Unit, University Hospital Virgen Macarena; 2 Mental Health Clinical Management Unit, University Hospital Virgen del Rocío; 3 Department of Psychiatry, University of Seville, Seville; 4 Faculty of Medicine and Health Sciences, University of Alcalá, Alcala de Henares; 5 Biomedical Research Networking Centre for Mental Health, CIBERSAM-ISCIII; 6 Ramón y Cajal Institute of Sanitary Research, IRYCIS, Madrid; 7 Mental Health Clinical Management Unit, University Hospital Virgen Macarena; 8 Biomedical Research Networking Centre for Mental Health, CIBERSAM-ISCIII , Seville, Spain

## Abstract

**Introduction:**

Age at onset of psychosis (AOP) is a key prognostic marker in psychotic disorders. Male gender, family history of psychosis, poor premorbid adjustment, cannabis use and a diagnosis of schizophrenia correlate with earlier AOP. However, the influence of environmental factors, such as urbanicity (a recognized risk factor for psychosis), has been less explored in relation to AOP.

**Objectives:**

To assess whether living in an urban or rural environment is associated with differences in AOP, and to determine whether this effect is independent of other sociodemographic and clinical factors.

**Methods:**

Data were drawn from the RedPEPSur study (ethics approval: SICEIA-2025-001567). The participants were aged 18–55, were treated at the Virgen del Rocío and Virgen Macarena University Hospitals (Seville, Spain), between 2021 and 2023, and were diagnosed with first-episode psychosis (FEP) according to ICD-10 criteria (F1x.4, F1x.5, F1x. 7, F20–29, F30.2, F31.2, F31.5, F31.6, F32.3, F33.3 and F53.1 codes). Rural was defined as municipalities with <30,000 inhabitants and <100 inhabitants/km². Cox regression estimated the effect of urban versus rural residence on risk of FEP onset at given ages, adjusting for male gender, family history of psychosis, poor premorbid adjustment, cannabis use and schizophrenia diagnosis. AOP was treated as survival time. Hazard ratios (HR) with 95% confidence intervals (CI) were reported. SPSS used; p <0.05 considered significant.

**Results:**

204 FEP patients were included; 125 (61.3%) male. Median AOP was 30 years (IQR: 22–39). 167 (81.9%) lived in urban areas; 68 (33.3%) had poor premorbid adjustment; 45 (22.1%) had family history of psychosis; 89 (43.6%) reported cannabis use; and 73 (35.8%) were diagnosed with schizophrenia. Urban residence was associated with significantly increased risk of earlier onset in FEP (HR = 1.47; 95% CI: 1.01–2.12; p = 0.043) in the multivariate Cox model. Predicted survival curves showing the effect of urban/rural living on the age at onset of psychosis are shown in Figure.

**Image 1:**
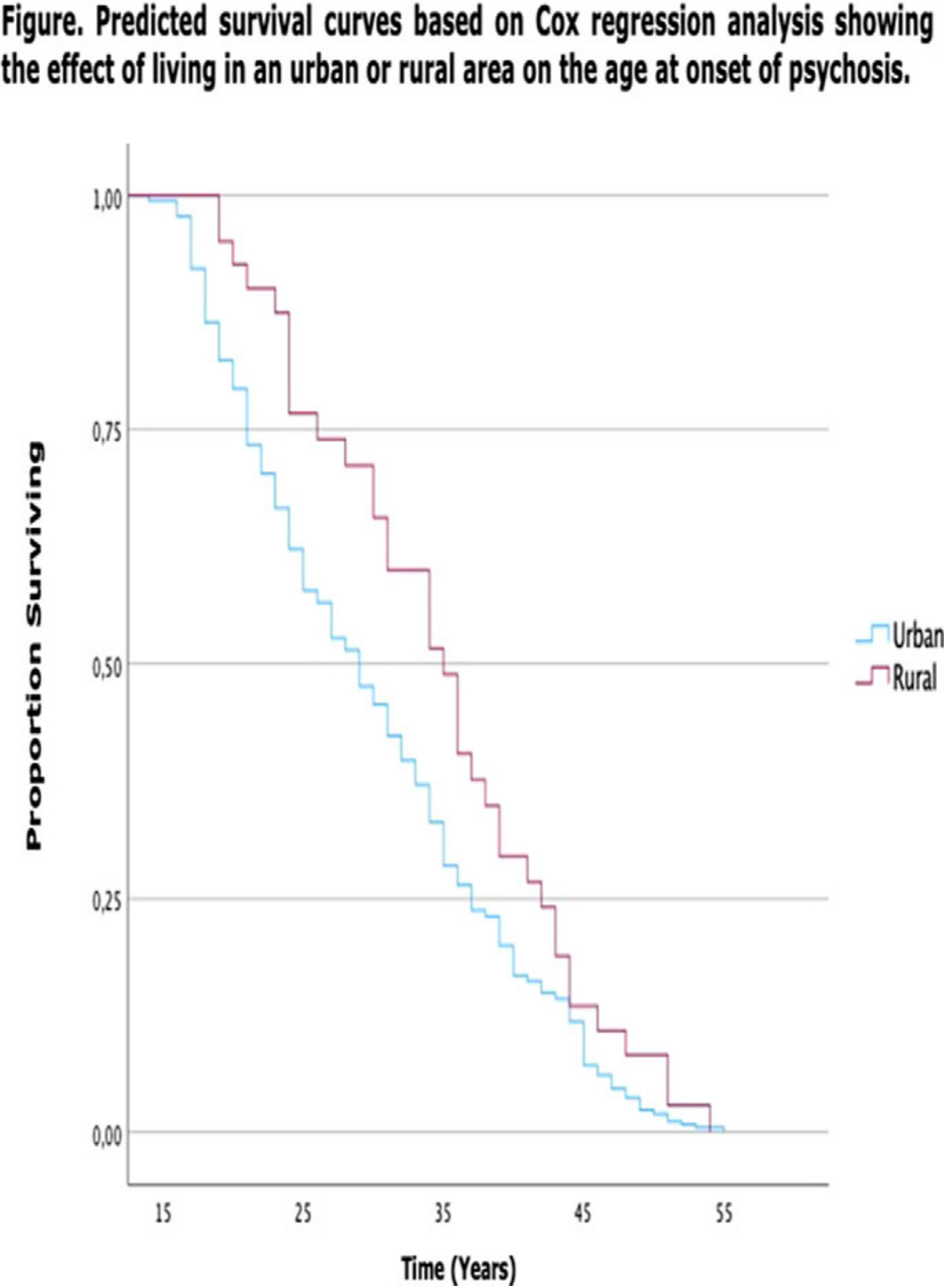
Image 1: Long description.

**Conclusions:**

In this clinical cohort, urban residence was independently and significantly associated with earlier AOP. Greater cumulative exposure to environmental risk factors in urban settings may underlie this association. Further studies employing granular environmental measures are needed to clarify the complex relationship between urbanicity and AOP.

**Disclosure of Interest:**

None Declared

## Long descriptions

### Image 1: Long description

The X-axis is labeled Time (Years) with increments at 15, 25, 35, 45, and 55. The Y-axis is labeled Proportion Surviving with values from 0,00 to 1,00 in 0,25 increments. A legend on the right identifies a light blue line as Urban and a maroon line as Rural. Both lines begin at 1,00 at year 15. The Urban curve shows a steeper, earlier decline, reaching the 0,50 survival mark at approximately 30 years. The Rural curve shows a more gradual decline, reaching the 0,50 survival mark later, at approximately 37 years. Both curves follow a step-function pattern, descending toward 0,00 as time approaches 55 years, with the Rural group maintaining a higher proportion of survival throughout most of the observed period.

Navigate back to Image 1:.

